# Human disturbance in riparian areas disrupts predator–prey interactions between grizzly bears and salmon

**DOI:** 10.1002/ece3.11058

**Published:** 2024-03-19

**Authors:** Megan S. Adams, Taal Levi, Mathieu Bourbonnais, Christina N. Service, Kyle Artelle, Heather Bryan, Paul Paquet, Trisalyn Nelson, Chris T. Darimont

**Affiliations:** ^1^ Department of Geography University of Victoria Victoria British Columbia Canada; ^2^ Raincoast Conservation Foundation Sidney British Columbia Canada; ^3^ Hakai Institute Campbell River British Columbia Canada; ^4^ Central Coast Indigenous Resource Alliance Campbell River British Columbia Canada; ^5^ Department of Fisheries and Wildlife Oregon State University Corvallis Oregon USA; ^6^ Department of Earth, Environmental and Geographic Sciences University of British Columbia Okanagan Kelowna British Columbia Canada; ^7^ Kitasoo Xai'xais Stewardship Authority, Kitasoo Xai'xais First Nation Klemtu British Columbia Canada; ^8^ School of Environmental Studies University of Victoria Victoria British Columbia Canada; ^9^ Department of Environmental Biology, and Center for Native Peoples and the Environment State University of New York, College of Environmental Science and Forestry Syracuse New York USA; ^10^ Department of Ecosystem Science and Management University of Northern British Columbia Prince George British Columbia Canada; ^11^ Department of Geography University of California Santa Barbara Santa Barbara California USA

**Keywords:** fragmentation, human disturbance, land‐use planning, *Oncorhynchus*, riparian habitat, *Ursus*

## Abstract

Wildlife must increasingly balance trade‐offs between the need to access important foods and the mortality risks associated with human‐dominated landscapes. Human disturbance can profoundly influence wildlife behavior, but managers know little about the relationship between disturbance–behavior dynamics and associated consequences for foraging. We address this gap by empirically investigating the consequences of human activity on a keystone predator–prey interaction in a region with limited but varied industrial disturbance. Using stable isotope data from 226 hair samples of grizzly bears (*Ursus arctos horribilis*) collected from 1995 to 2014 across 22 salmon‐bearing watersheds (88,000 km^2^) in British Columbia, Canada, we examined how human activity influenced their consumption of spawning salmon (*Oncorhynchus* spp.), a fitness‐related food. Accounting for the abundance of salmon and other foods, salmon consumption strongly decreased (up to 59% for females) with increasing human disturbance (as measured by the human footprint index) in riparian zones of salmon‐bearing rivers. Declines in salmon consumption occurred with disturbance even in watersheds with low footprints. In a region currently among the least influenced by industrial activity, intensification of disturbance in river valleys is predicted to increasingly decouple bears from salmon, possibly driving associated reductions in population productivity and provisioning of salmon nutrients to terrestrial ecosystems. Accordingly, we draw on our results to make landscape‐scale and access‐related management recommendations beyond current streamside protection buffers. This work illustrates the interaction between habitat modification and food security for wildlife, highlighting the potential for unacknowledged interactions and cumulative effects in increasingly modified landscapes.

## Graphical abstract

We investigated potential drivers of salmon consumption by bears in interior and coastal watersheds that varied in levels of disturbance. We found that human footprint in riparian areas of salmon‐bearing watersheds affected bear diets more than the amount of salmon biomass available, showing that human activity can disrupt an otherwise strong predator–prey association.

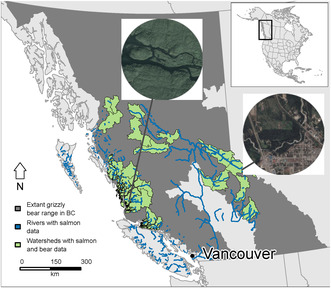

## 1 INTRODUCTION

Theory and evidence to guide managers on the response by wildlife to gradients of habitat disturbance present an increasingly divergent picture. Biodiversity losses are most likely to occur at high levels of habitat loss and fragmentation (Newbold et al., 2016; Ramírez‐Delgado et al., 2022). Emerging evidence, however, suggests that the rate of impacts to and losses of biodiversity may be the greatest when disturbance occurs in relatively intact landscapes (Betts et al., 2017; Watson et al., 2018). Although mechanisms behind the disproportionate impacts of early stages of human disturbance are unclear, direct effects of human‐caused mortality can cause population declines, while behavioral effects (e.g., fear and avoidance) can elicit multiple ecological effects at the population and community levels. Particularly for large‐bodied vertebrates, humans account for more adult mortality than any other predator (Darimont et al., 2015; Estes et al., 2011). The probability of mortality is particularly acute in areas of human disturbance, where the footprint of roads, development, and infrastructure (collectively ‘human footprint’; Venter et al., 2016) increases exploitation, conflict, and transportation‐induced mortality of wildlife (Hill et al., 2020; Morales‐González et al., 2020; Torres et al., 2016).

In addition to direct mortality, the perception of mortality risk can lead to behavioral changes. These include spatial or temporal avoidance of human activity (Gaynor et al., 2018; Palmer et al., 2022; Tucker et al., 2018). Although mortality can be reduced by wildlife management, confronting potential effects of behavioral avoidance requires additional consideration given the persistent nature of infrastructure and development on the landscape (Crooks et al., 2017). Specifically, avoidance of persistent risk by wildlife could lead to population declines if fitness is reduced by restricted access to resources important for reproduction and survival (Laundré et al., 2010; Lima & Bednekoff, 1999).

The keystone interaction between grizzly bears (*Ursus arctos horribilis*) and Pacific salmon (*Oncorhynchus* spp.) provides an opportunity to examine how avoidance of human activity could negatively affect the behavior of species and community interactions via decreased access to key foods. Salmon offer high‐quality nutritional resources for bears (Hilderbrand et al., 1999), which, unlike other important foods, are spatially clustered (Schindler et al., 2003). Moreover, increased salmon consumption is linked to measures of fitness including larger body mass, increased litter sizes, and higher population densities (Bryan et al., 2013; Hilderbrand et al., 1999). Greater densities of salmon‐supported bear populations can also then increase seed dispersal of berry species via bear feces, which in turn increases food supply for berry eaters in future years, including bears themselves (Harrer & Levi, 2018; Levi et al., 2020; Shakeri et al., 2018). Finally, partially consumed salmon carcasses deposited by bears in riparian and forest environments provide plant and animal communities with marine‐derived nutrients (Hocking & Reynolds, 2011; Schindler et al., 2003).

Despite these benefits to bears and the broader environment, accessing salmon can expose bears to risk. Along valley bottoms, where bears congregate to forage on salmon, humans often have substantial and persistent activities and infrastructure associated with human‐caused mortality (McLellan et al., 2018; Morales‐González et al., 2020; Mowat et al., 2013). If bears respond behaviorally by foregoing feeding on salmon, particularly at the initial stages of human disturbance, then continued development could increasingly threaten the food security of bears. Reduced foraging on salmon would also undermine the keystone bear–salmon interaction that is responsible for fertilizing forests and feeding invertebrate and vertebrate scavengers with the remains of salmon carcasses (Levi et al., 2020). Whereas fish‐bearing streams are protected with fixed‐width buffers (generally ranging from ~15 to 30 m) through various regional, state/provincial, and federal legislation in the Pacific Northwest of Canada (Canada, 2019; Province of British Columbia, 2016, 2018; Richardson et al., 2012), adjacent riparian areas and valley bottoms are vulnerable to degradation from human activity (Lee et al., 2004; Waples et al., 2009).

To understand how habitat and food security might interact as conservation concerns for wildlife, we used stable isotope analysis to estimate the proportion of salmon in annual diets of grizzly bears across a gradient of human disturbance in British Columbia (BC), Canada. We assessed the association between human footprint (Venter et al., 2016) and salmon consumption at a watershed scale, accounting for salmon biomass density, salmon species diversity, and proxies for alternative foods (precipitation, temperature, and early‐seral forest). Our results and recommendations can guide land‐use planning by Indigenous, provincial, and federal managers responsible for bear–salmon–human systems. In a region among the least adversely affected by industrial activity on earth, this work illustrates potential adverse impacts of encroaching human activity on predator–prey interactions.

## 2 METHODS

### 2.1 Study area and sample collection

We used stable isotope signatures of hair samples (*n* = 226 individual‐year combinations) to estimate annual diets of individual grizzly bears in salmon‐bearing watersheds across BC (see below; Adams et al., 2017). Isotopic data from some of these samples first appeared in Mowat and Heard (2006) (*n* = 36 males, *n* = 14 females), collected across BC from 1995 to 2003. We augmented these data with hair from coastal grizzly bears (*n* = 135 males, *n* = 41 females) that we collected from 2009 to 2014, for a total of 158 unique individuals (Adams et al., 2017). We determined sex, species, and individual identity using information from seven microsatellite loci plus a sex marker from hair samples (Wildlife Genetics International, Nelson, BC). In cases where coastal individuals were sampled in multiple locations within the same year, we attributed the individual's salmon consumption value to the sample location that yielded the most guard hairs (ensuring a sufficient quantity for preparation of samples for isotopic analyses). Intra‐annual re‐detections all occurred within the same watershed of detection. When individuals were detected in multiple years, we considered each bear‐year case separately (*n* = 68 recaptured individuals, ranging from two to five recaptures across years) (Adams et al., 2017).

We conducted coastal research in the traditional territories of the Haíɫzaqv (Heiltsuk), Kitasoo/Xai'xais, Nuxalk, and Wuikinuxv Nations, who were also partners in this work. Non‐invasive hair collection from coastal grizzly bears was approved by the Animal Care Committee at the University of Victoria (permit no. 2012‐018), following applicable requirements concerning animal care and wildlife research (Canadian Council for Animal Care; Field et al., 2019; Sikes & Gannon, 2011). We also had a permit (no. 106703) from BC Parks to sample in conservancies. Research protocol agreements with partnering Indigenous governments prohibit us from displaying sample locations.

**FIGURE 1:**
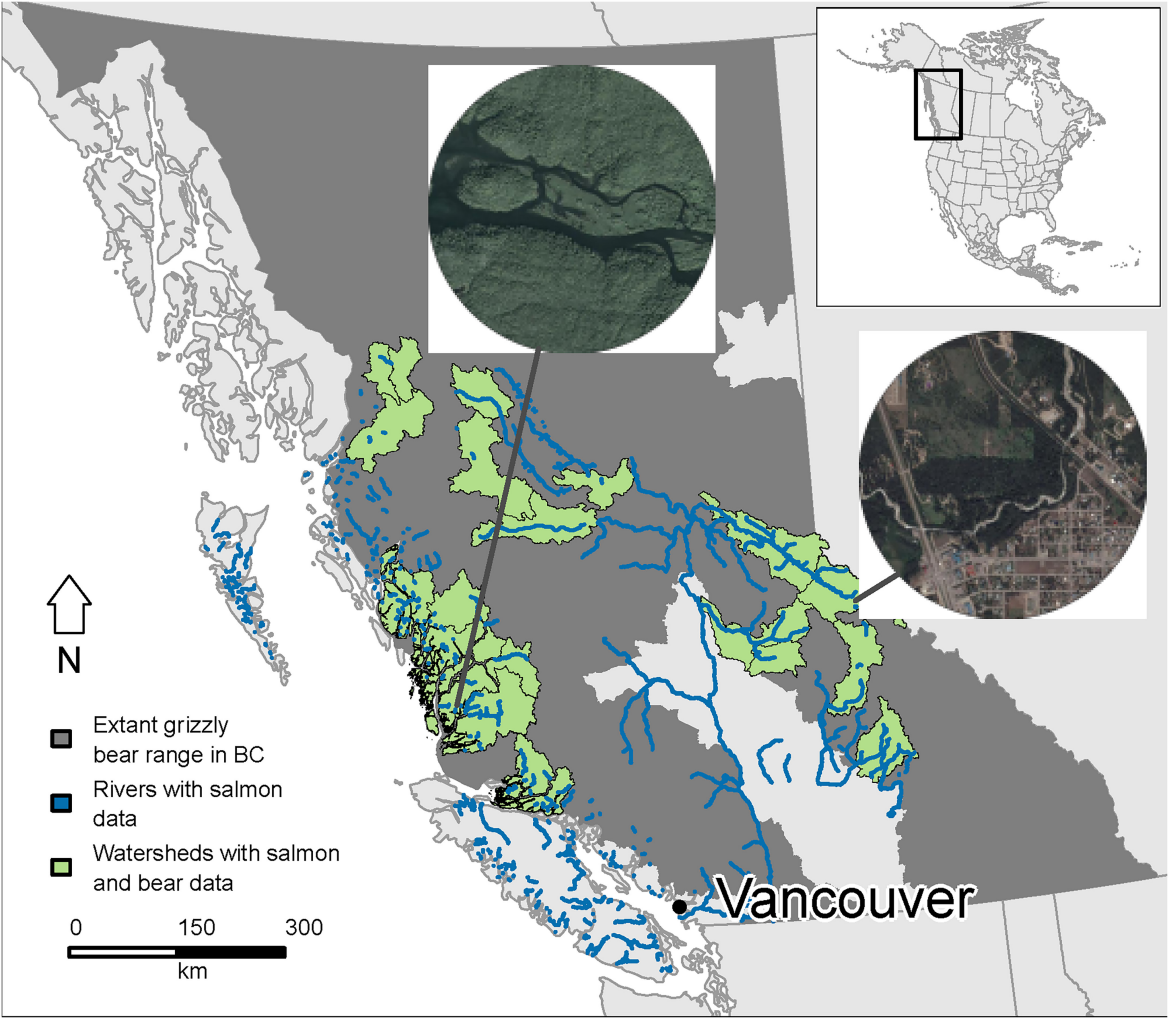
Third‐order watersheds assessed (*n* = 22, in light green) within extant range of grizzly bears (*Ursus arctos horribilis*, in dark gray) in British Columbia, Canada. Salmon‐spawning reaches of rivers with enumeration data available are displayed in blue. Aerial photographs show human footprint in riparian areas in watersheds in lower (HFI ≈ 0.05) and upper (HFI ≈ 10) quantiles of the HFI covariate.

We linked bear samples to salmon biomass data (see below), only considering hair samples collected within watersheds for which there were also salmon enumeration data (*n* = 22, covering ~88,000 km^2^ in Figure 1; see full details in Appendix S1: Sections 1 and 3). We used third‐order basins to define watersheds as delineated by the Strahler stream order hierarchy of tributaries (Figure 1; Province of British Columbia, 2011) as the spatial unit for aggregating predictor variables for detected individuals. We excluded areas of unsuitable habitat, including water, rock, and ice (Artelle et al., 2016). Useable portions of watersheds ranged from 1459 to 7674 km^2^, with a median of 3392 km^2^. For comparison, the average home range areas for individual grizzly bears are generally an order of magnitude smaller (Table S1), reducing the probability that individual bears spanned multiple third‐order watersheds in a given year. Watersheds varied in salmon abundance, both in terms of the biomass and diversity of salmon species available (single species to five species) and length of spawning reach within the watershed (Figure 1).

The landscape and presence of human activity varied within selected watersheds of the study area. Whereas coastal watersheds generally have low human population densities and are generally without extensive or connected road networks (Service et al., 2018), interior watersheds to the east of the Coast Mountain range have a relatively higher presence of human population density (e.g., more settlements with higher population densities) and ongoing industrial activity adjacent to salmon‐bearing rivers (Figure S1) and throughout the watersheds in general (see Figure S2; Section 2.3.3 below). We note that grizzly bears have been extirpated in areas of British Columbia adjacent to large urban centers with moderate‐to‐high human footprints (e.g., Vancouver and the lower drainages of the Fraser River and surrounding areas; Figure 1).

### 2.2 Estimating salmon consumption

We used hair collected during the shedding phase of the annual molt (May–June) to assess diet of grizzly bears (see Appendix S1: Section 1.1). This ensured that isotopic measures represented the assimilated diet during the entire previous year's hair growth (Hilderbrand et al., 1999). We prepared and processed hair samples for stable carbon (δ^13^C) and nitrogen (δ^15^N) isotope ratio estimation via gas chromatography–mass spectrometry (University of Saskatchewan, Saskatoon, SK, Canada; Service et al., 2018). We estimated annual dietary contributions with Bayesian mixing models that use δ^15^N and δ^13^C in consumer and food resource samples, trophic discrimination values, and associated uncertainties to predict the proportion of a diet comprising a given food resource (Moore & Semmens, 2008) (see Appendix S1: Sections 1.2–1.7, Tables S2 and S3). We used the median posterior value across the distribution of model estimates of salmon consumption in the annual diet of each individual (measured as a proportion of relative contribution, hereafter ‘salmon consumption’) as the response variable in analyses (Adams et al., 2017; Moore & Semmens, 2008; Service et al., 2018). Observed salmon consumption as a relative proportion of total annual diet ranged from 0.03 to 0.95.

### 2.3 Potential covariates affecting salmon consumption

We modeled spatially and temporally explicit covariates expected to influence annual salmon consumption using a set of a priori mixed‐effects beta regression models (see Section 2.4 below, Table S4). For each watershed, we accounted for geographic and temporal variation in salmon availability, alternative food availability, and human footprint (Figures S1 and S2). We considered each of these factors from the year before hair collection, because hair‐derived isotopic information relates to the preceding year's growth.

#### 2.3.1 Salmon availability

We used a geo‐referenced database containing annual abundance estimates of the five Pacific salmon species (*Oncorhynchus tshawytscha*, *O*. *kisutch*, *O*. *nerka*, *O*. *gorbuscha*, and *O*. *keta*) across their spawning reaches in BC (FOC, 2016) to estimate annual biomass density (‘biomass’) and species diversity (‘diversity’) present in each watershed (see Appendix S1: Section 2). Estimates from regularly monitored streams, however, are frequently deficient (Price et al., 2017). Accordingly, we employed an imputation method for missing data points for a given species, year, and watershed developed for this dataset (Bryan et al., 2014). We then calculated salmon biomass in each watershed by multiplying abundance estimates by the average mass of salmon (kg) and summing across species, based on average mass of both sexes and a 1:1 sex ratio (Bryan et al., 2014). We divided total biomass by the length of spawning area of each salmon‐bearing stream contained within the watershed and summed biomass if multiple salmon streams existed within the watershed. Finally, we divided the total biomass estimate by the area of the watershed to compute an estimated salmon density within watersheds. Complementing these estimates of abundance, we used the Shannon diversity index to estimate salmon diversity (‘diversity’), weighting the number of salmon species present in each watershed by the proportion of the total estimated salmon biomass comprising each species (Service et al., 2018). Increased species diversity results in spatiotemporal asynchrony of salmon availability that we reasoned would extend foraging opportunities for bears (Service et al., 2018).

The effort, and therefore reliability, of salmon abundance measurements in BC has eroded over time and is variable across watersheds and regions (Price et al., 2017). We explored the potential influence of varying enumeration effort and proximity to the coast on model fit in post‐hoc model diagnostics (see Appendix S1: Section 3).

#### 2.3.2 Alternative food availability

In addition to salmon, grizzly bears consume emergent vegetation, berries, roots, and ungulates within the study area (Adams et al., 2017). We used growing season climatic variables and area of early‐seral forest as proxies for alternative food availability (Artelle et al., 2016; Mowat et al., 2013) because direct and comprehensive spatial and temporal estimates of plant and ungulate availability across BC do not exist. We used mean annual spring and summer (April–September) temperatures (°C) and summer (May–September) precipitation (mm) as proxies for plant food abundance (Artelle et al., 2016). We used the program *Climate BC*, which assembles climatic variables gathered from weather stations across BC to an 800 m × 800 m resolution with high accuracy (*R* ^2^ > .9 for most predicted and weather station measurements; Wang et al., 2015). We created a 4 km × 4 km grid of points within useable habitat across each watershed to extract temperature and precipitation data. We averaged data over all points within the watershed for a given year.

Regenerating early‐seral forest supports high densities of foraging resources for grizzly bears, such as emergent vegetation and berries, deer (*Odocoileus* spp.), and moose (*Alces alces*), compared with mature forest stands (Fisher & Wilkinson, 2005). However, for areas disturbed greater than 10 years, canopy cover is likely to reduce herbaceous growth that attracts ungulates (Swanson et al., 2011). Accordingly, we used a composite dataset of landscape disturbance developed using Landsat imagery (White et al., 2017) to quantify the proportion of annual area of early‐seral habitat within a watershed (‘early‐seral’). We classified early‐seral stage habitat as fire disturbances and forest harvest blocks between one and ten years of age, relative to the date of bear hair collection. We note that this variable does not account statistically for disturbance from human footprint in riparian buffers, which we assessed independently (see below).

#### 2.3.3 Human footprint

We used the global human footprint index (HFI; Venter et al., 2016) as a proxy for the effects of human influence on riverine habitats. The HFI characterizes the extent of built environments from multiple proxies of human influence (e.g., population density, nighttime lights, infrastructure, agriculture, roads, navigable waterways, and railways), which were all scaled and weighted according to relative levels of human pressure (Venter et al., 2016). The full HFI dataset ranged from 0 (natural environments; e.g., unlogged regions of coastal temperate rainforest) to 50 (urban environments; e.g., the city of Vancouver, BC). Composite HFI datasets were compiled for 1993 and 2009 (Venter et al., 2016). We assigned human footprint data depending on temporal proximity to the HFI compilations. Specifically, bears detected from 1993 to 2001 were assigned data from the 1993 HFI map (Sanderson et al., 2002), whereas bears detected from 2002 to 2014 were assigned data from the 2009 HFI map (Venter et al., 2016). Assigned HFI data were always within an eight‐year buffer of the relevant HFI compilation. We note that change to human footprint occurs relatively slowly, making these two datasets highly temporally correlated (Venter et al., 2016) and with minimal changes occurring within the timescale of our analysis.

We considered the density of human footprint within riparian zones of all potential spawning salmon reaches within each watershed (Province of British Columbia, 2006) using a 1000 m buffer on either side of the river system as a riparian zone estimate, where bear activity during the spawning season is generally concentrated (Figure S1; Titus & Beier, 1999). We summarized mean HFI (‘footprint’) for all riparian zones of reaches within each study watershed (ranging from 0.00 to 10.4).

We note that the HFI data are coarse in resolution and might miss temporary resource roads for energy, mining, forestry, and other industrial activities that impact the behavior and movement of bears. Available datasets for British Columbia that cover some, but not all, of these roads (Province of British Columbia, 2017) have no temporal component. Accordingly, for each bear diet‐year considered, it would be impossible to determine when roads were built, in use, or deactivated. Given the large spatial scale of our bear diet dataset, as well as literature that supports the human footprint dataset as a proxy for human disturbance for wildlife (Tucker et al., 2018; Watson et al., 2018), we thus used the HFI data in our analysis.

### 2.4 GLMM analyses

We performed all analyses in R 3.2.1 (R Development Core Team, 2018). We used a generalized linear mixed model (GLMM; Bolker et al., 2009) and information‐theoretic model selection framework (Burnham & Anderson, 2002) to identify associations among patterns of salmon consumption and salmon availability, alternative terrestrial foods, and human footprint covariates. We developed a candidate model set from combinations of predictor variables and interactions among them that we considered ecologically plausible for predicting salmon consumption by grizzly bears (Table S4). To account for the proportional and continuous nature of annual contribution of salmon to diet, we assumed the response variable was beta‐distributed (Fox, 2015; Moore & Semmens, 2008). To place predictor variables on a common scale and to allow a direct comparison of their effect sizes, we centered and scaled continuous variables by two standard deviations before inclusion in models (Gelman, 2008; Schielzeth, 2010). We fit GLMMs using the *glmmADMB* package (Skaug et al., 2013). We used a logit link function with intercept‐only random effects for year (to account for temporal variation) and watershed (to account for spatial variation). We ranked models using Akaike's information criterion, correcting for small sample sizes (Bolker et al., 2009; Burnham & Anderson, 2002). We assessed the strength of predictor variables by comparing parameter estimates of the top models.

## 3 RESULTS

**FIGURE 2:**
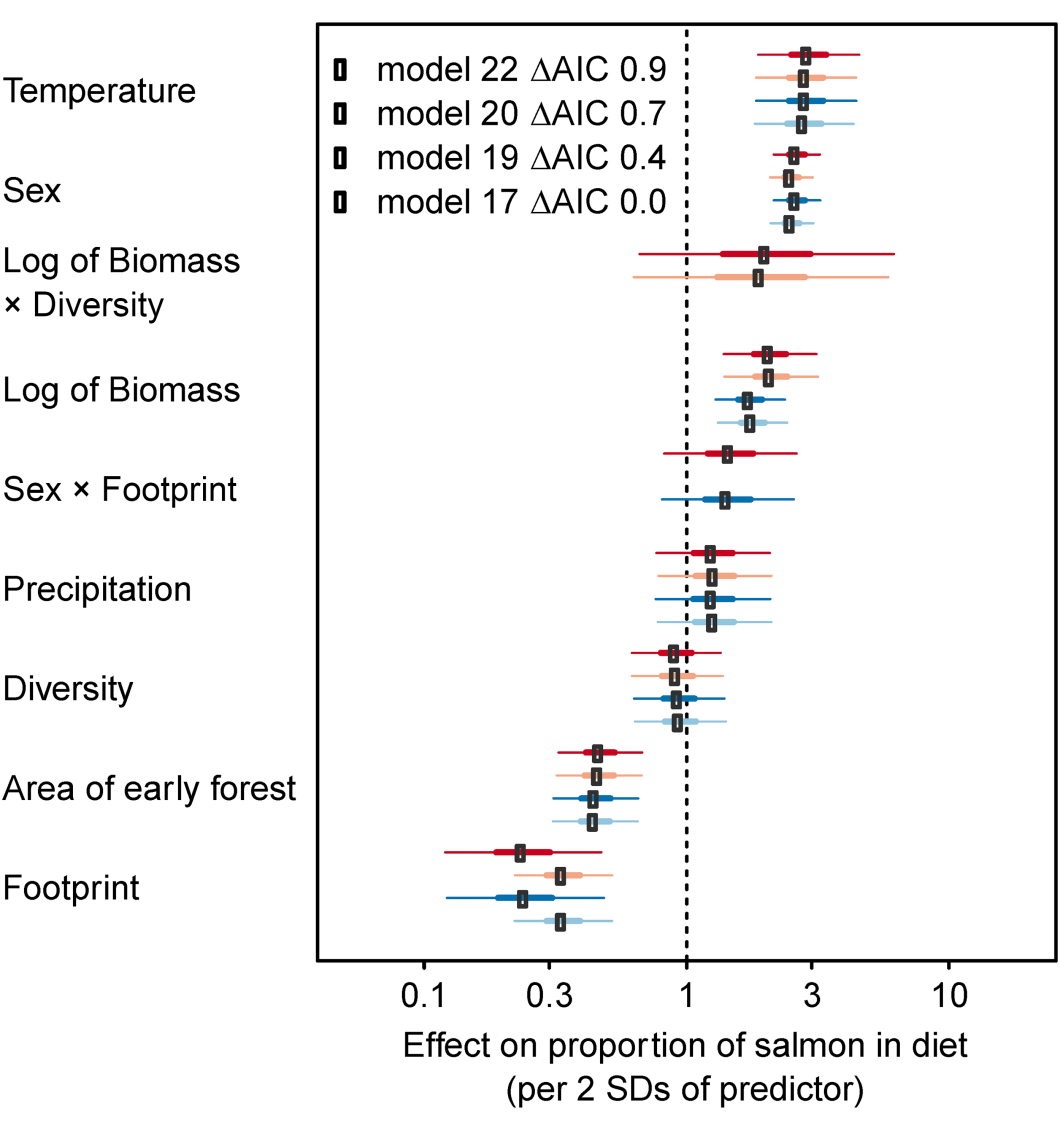
Effect sizes of covariates associated with salmon consumption by grizzly bears (*Ursus arctos horribilis*; *n* = 226) across 22 watersheds in British Columbia, 1995–2014. We centered and scaled continuous variables by two standard deviations before inclusion in models (Gelman, 2008; Schielzeth, 2010) in order to place predictor variables on a common scale and to allow a direct comparison of their effect sizes. Dots represent parameter estimates as odds ratios. Thick and thin bars represent 50% and 95% confidence intervals, respectively, from the top four models (see model structures in Table 1 and Table S4; model 17 is most parsimonious). Units are in two standard deviations of each predictor. Parameters are ordered by mean effect size. The least parameterized model had the lowest AIC, indicating lack of support for more complex models.

**FIGURE 3:**
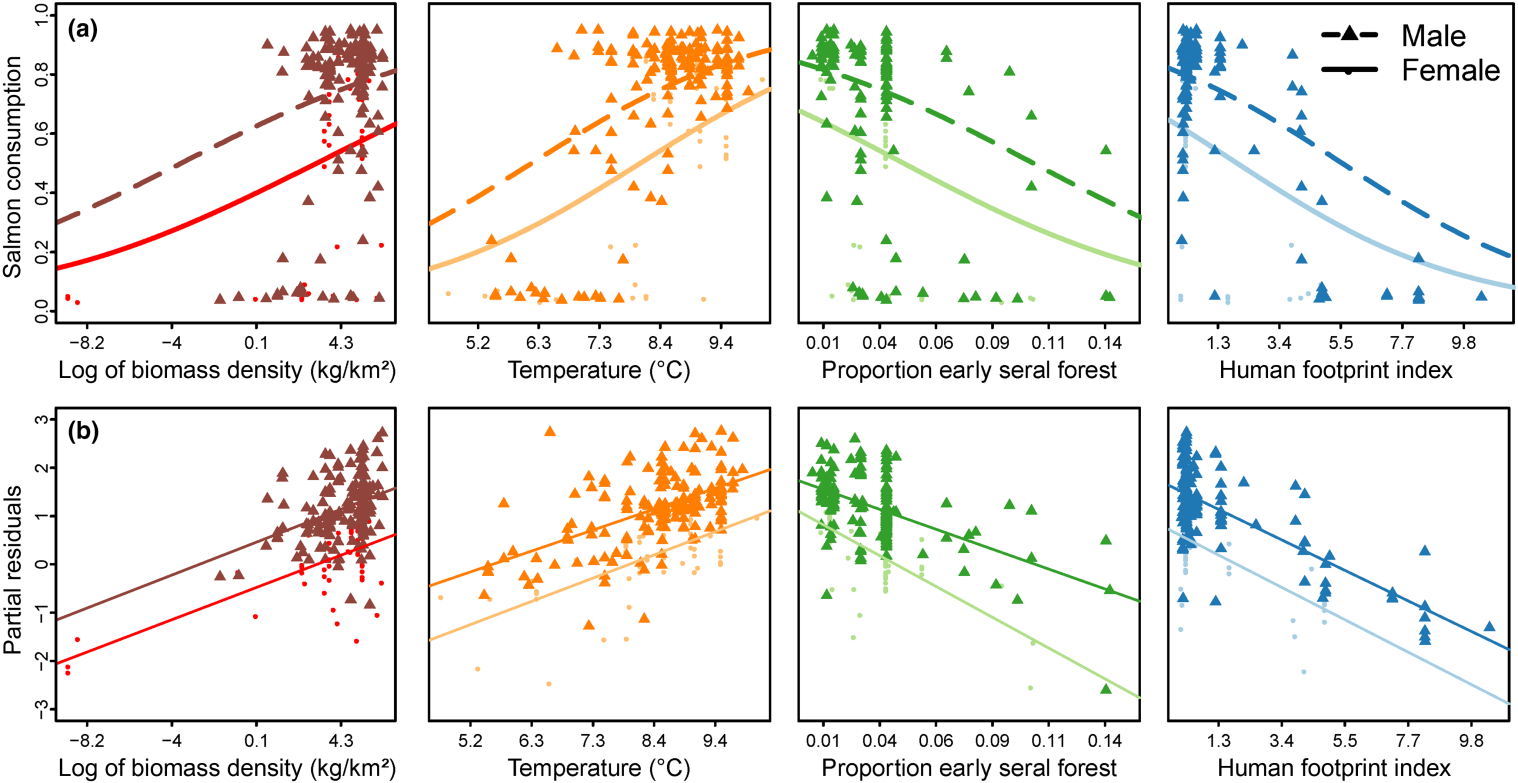
Associations between salmon consumption as proportion of total annual diet of grizzly bears (*Ursus arctos horribilis*; *n* = 226) and several covariates across 22 watersheds in British Columbia, 1995–2014. (a) Log of salmon biomass density, mean annual spring and summer temperatures, proportion of early forest area, and riparian human footprint index in watersheds are shown for the simplest top models (model 17), for each sex. Each point represents raw data associated with each factor level, not accounting for any relationships in the top model (including the covariate modeled on the *x*‐axis), whereas (b) partial residual plots demonstrate the effect and fit of each covariate when accounting for all others. Positive salmon consumption at zero salmon biomass reflects salmon enumeration data only covering an index subset of all salmon‐bearing waterways.

**FIGURE 4:**
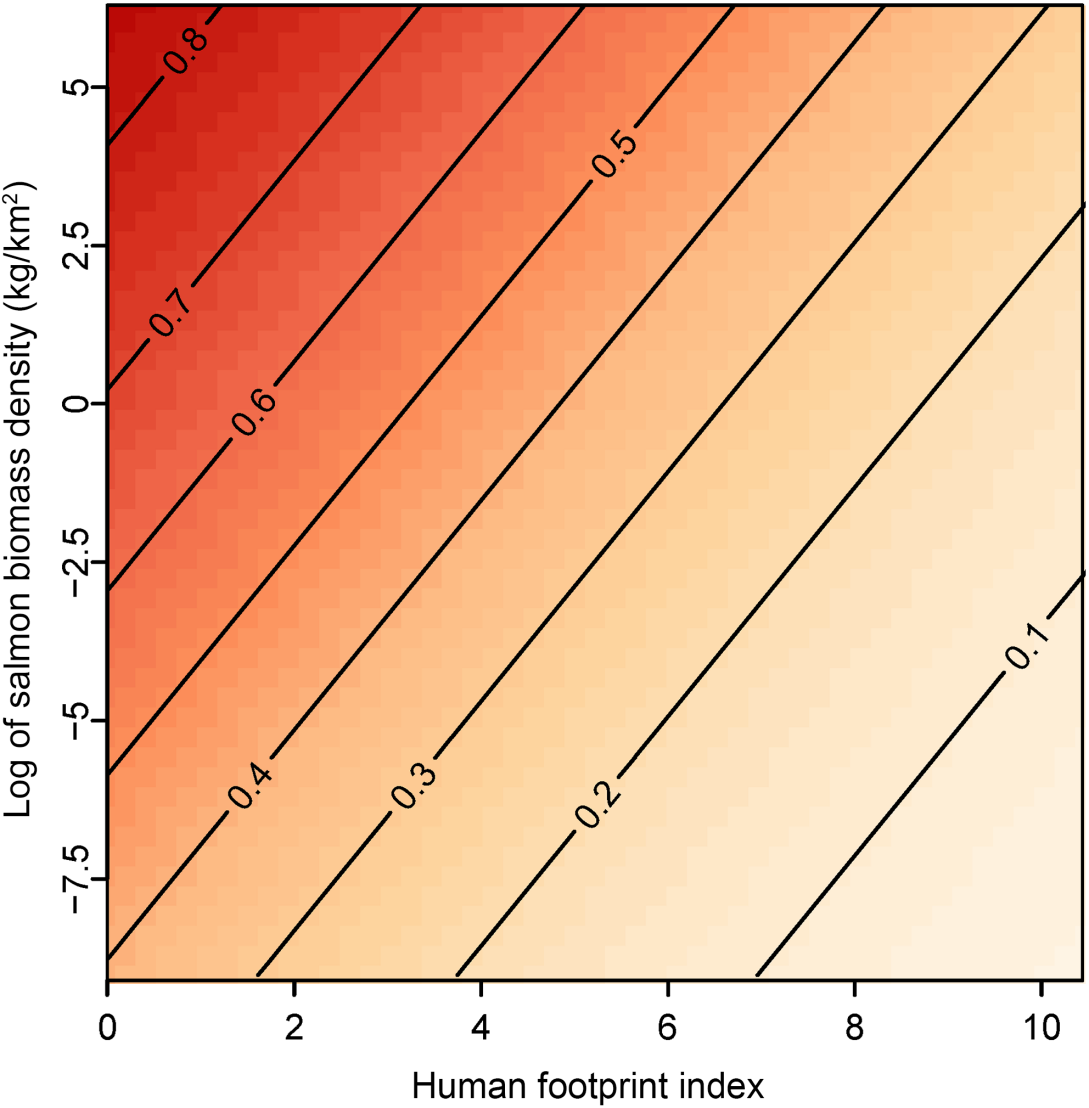
Predicted salmon consumption as proportion of total annual diet (*z*‐axis) of grizzly bears (*Ursus arctos horribilis*) across 22 watersheds in British Columbia, 1995–2014, decreases with increasing human footprint (*x*‐axis) at a greater rate compared with the reduction of salmon abundance (*y*‐axis). Model predictions for salmon consumption are positive at near‐zero salmon biomass density because salmon enumeration data only cover a subset of all salmon‐bearing waterways.

We identified one top model among 25 competing models, but we include results for three additional less parsimonious models that were within ∆AIC < 2.0 so that the effect sizes for unsupported hypothesized predictors can be visualized (Table S4). Salmon consumption was inversely associated with human footprint in riparian zones (1000 m buffers around spawning reaches, Figures 2 and 3). Standardized coefficient estimates suggest that the negative association with human footprint had the greatest influence on consumption of all predictors tested and was stronger than the positive association with salmon biomass density (Figures 2, 3, 4). All top models supported sex as an important predictor; males consumed more salmon than females. We did not find support of an interaction term between sex and footprint, indicating that male and female consumption of salmon declined similarly with increasing footprint (see outputs for models 19 and 22, noting females as the base condition for sex; Figure 2, Table 1). Additionally, in all top models, salmon consumption was greater in areas with higher salmon biomass density and higher temperature (Figures 2 and 3). Salmon consumption was moderately lower with increased proportions of early‐seral forest in the watershed (Figures 2 and 3). Interaction terms between salmon abundance and footprint did not improve model predictions (Table S4). We found little to no effects of salmon diversity or precipitation on salmon consumption in any top models (Figure 2, Table 1).

**TABLE 1:** Parameter estimates (with 95% confidence intervals, given as ±−2·SE) for four GLMMs with ΔAIC < 2 (Table S4) used to predict annual proportion of dietary salmon in diets of grizzly bears (*Ursus arctos horribilis*) across BC (1995–2014).

	Model 17	Model 19	Model 20	Model 22
Intercept	0.170 (−0.152, 0.493)	0.133 (−0.196, 0.463)	0.133 (−0.193, 0.458)	0.090 (−0.242, 0.422)
Sex	**0.923** (0.736, 1.111)	**0.968** (0.764, 1.171)	**0.920** (0.733, 1.107)	**0.967** (0.764, 1.170)
Log of biomass	**0.579** (0.273, 0.884)	**0.558** (0.253, 0.862)	**0.742** (0.331, 1.152)	**0.731** (0.324, 1.138)
Diversity	−0.054 (−0.454, 0.345)	−0.066 (−0.462, 0.331)	−0.081 (−0.478, 0.316)	−0.091 (−0.482, 0.299)
Footprint	**−1.081** (−1.508, −0.655)	−**1.416** (−2.105, −0.727)	**−1.081** (−1.507, −0.655)	**−1.434** (−2.119, −0.750)
Temperature	**1.032** (0.597, 1.467)	**1.049** (0.609, 1.488)	**1.049** (0.611, 1.487)	**1.071** (0.628, 1.514)
Precipitation	0.244 (−0.252, 0.741)	0.231 (−0.272, 0.734)	0.248 (−0.245, 0.741)	0.232 (−0.266, 0.730)
Early‐seral	**−0.802** (−1.174, −0.429)	**−0.797** (−1.169, −0.425)	**−0.766** (−1.138, −0.395)	**−0.757** (−1.126, −0.389)
Sex × footprint	–	0.360 (−0.219, 0.940)	–	0.385 (−0.196, 0.966)
Log of biomass × diversity	–	–	0.653 (−0.465, 1.772)	0.703 (−0.414, 1.819)

*Note*: The contrast level for sex was female. Continuous predictors were centered (mean subtracted) and scaled (divided by 2 SD). Bold values indicate estimates with confidence intervals that did not overlap zero; parameter estimates are log odds.

Using coefficient estimates from our most parsimonious top model (Figure 2 and Table 1), we estimated how the annual proportion of salmon consumption was associated with variation in the salmon biomass and footprint covariates. We predicted consumption by males and females separately across observed variation in salmon biomass density, holding all other covariates at their mean. For watersheds with lower salmon biomass (10% quantile, ~10.8 kg/km^2^), predicted annual proportion of salmon in diets of males and females was 0.70 and 0.48, respectively, whereas in watersheds with higher salmon biomass (90% quantile, ~286.5 kg/km^2^), salmon consumption was higher by roughly 13% and 28% (i.e., 0.78 and 0.59), respectively.

The influence of HFI was the most pronounced among the predictors. Holding salmon biomass and other predictors at their mean values, the predicted proportion of salmon in annual diets of males and females was 0.80 and 0.62 in watersheds least affected by human footprint (5% quantile, HFI = 0.05), but was lower by roughly 42% and 59%, respectively (i.e., to 0.46 and 0.25), in watersheds most affected by human footprint (95% quantile, HFI = 6.2). Notably, this range of footprints represents relatively low impact on the landscape, with the upper quantile of footprint in our analysis (HFI = 10.5) characterizing limited infrastructure about one‐tenth of the maximum footprint worldwide score of 50 (i.e., urban environments).

## 4 DISCUSSION

Although salmon offer a profitable, spatially constrained, and otherwise safely acquired resource, our results suggest that grizzly bears reduced consumption amidst even low levels of human footprint in salmon‐bearing streams. Existing riparian management regulations, designed to protect salmon and their in‐stream habitats, thus appear insufficient to mitigate this disturbance. Interaction terms between salmon abundance and footprint did not strengthen model predictions (Table S4), suggesting bears avoid disturbance similarly at all levels of salmon biomass availability. Combined, these patterns suggest that bears may forgo profitable fitness‐related foods if perceived risks from human activity are too high.

Such behavior might have population‐scale implications. The 59% reduction in salmon consumption by females across the range of footprints considered here is expected to correspond to reduced litter sizes and population densities, given the observed relationships between salmon consumption and measures of population productivity across populations (Hilderbrand et al., 1999). Additionally, although the mechanism remains unknown, our results suggest a disturbance‐mediated reduction in salmon consumption that has occurred during a short period of large‐scale habitat degradation over the past two centuries relative to the millennia in which bears and people have coexisted. Accordingly, we might expect lower populations of bears now compared with periods prior to industrial activity. Owing to a lack of available data at the spatial and temporal scales of our analyses, our work did not incorporate estimated bear densities, so as to account for potential influence of intraspecific competition. We do, however, account for the sex of bears. Work in other bear–salmon systems demonstrates the strong effect sex and competition play in determining access to salmon, particularly for females with cubs (Rode et al., 2006).

Overall, we found mixed support related to the association of salmon consumption with the abundance of foods. Salmon consumption increased with salmon biomass but not species diversity, differing from recent findings at smaller spatial scales that showed how species diversity affects foraging by black bears (*U*. *americanus*) (Service et al., 2018). Our failure to detect an association might relate to many interior watersheds hosting only sockeye salmon (*O*. *nerka*). Intraspecific measures of diversity for ecologically and genetically distinct ‘conservation units’ (Canada, 2005) could be more relevant to future research in interior regions. Notably, we also lack population‐level phenological data for salmon, which, for large single‐species runs such as sockeye, prolongs foraging opportunities and increases salmon consumption by bears (Deacy et al., 2018; Nesbitt & Moore, 2016; Schindler et al., 2013). We also note that due to the large spatial and temporal scales of our analyses, we were unable to predict how catchability of salmon within watersheds (e.g., depth and flow of rivers, density of salmon within a given reach, and capture rates by bears; Gende & Quinn, 2004) could underlie variation in salmon consumption by bears. We found modest associations, however, with estimates of alternative foods. In watersheds with more early‐seral forests (a proxy for plant and herbivore biomass), bears consumed less salmon, suggesting higher use of alternative foods that are spatially dispersed and thus less‐risky resources. Contrary to predictions, salmon consumption was positively associated with temperature, our other proxy for abundance of alternative foods. We postulate that higher temperatures across habitable areas of watersheds could cause lower river levels associated with lower body condition or higher mortality in migrating salmon (Rand et al., 2006), which could make an equivalent spawning biomass more available to bears as fish might be easier to catch. Higher temperatures are also associated with coastal areas (Figure S2) where many small salmon streams are not enumerated. Thus, the positive association with high temperature may be a proxy for the availability of unenumerated salmon. Alternatively, salmon consumption may be higher in warmer, coastal areas if salmon are more accessible to bears compared with watersheds in the interior with cooler temperatures.

Accounting for estimates of alternative foods, the pattern we detected of reduced salmon consumption with higher measures of HFI occurred in watersheds where human activities will likely continue to alter landscapes and are set against a background of multiple stressors to bear–salmon interactions. Habitat destruction via industrial agriculture, urban development, forestry, and oil and gas activities in remaining grizzly ranges in BC is predicted to rapidly expand over the coming decades (Shackelford et al., 2018). Such disturbance may impact salmon‐spawning habitat while also reducing salmon foraging activity by bears (Armstrong et al., 2019; Schindler et al., 2003). In addition to development, climate change may disrupt bear–salmon interactions. Recent work in Alaska showed that in warmer years, bears consumed fruit (elderberries; *Sambucus racemosa*) preferentially over sockeye salmon when both were available (Deacy et al., 2017). Together, development and climate change are expected to reduce both salmon availability and the time that bears spend foraging, with downstream consequences on ecosystem processes that depend on bears for provisioning of salmon carcasses.

In spite of both spatial and temporal coarseness, our data spanning two decades and a 22‐watershed landscape scale offer insight into potential concerning impacts of habitat disturbance and support consideration of a more precautionary approach to land‐use planning and habitat alteration. Specifically, the patterns we observed suggest that existing guidelines and enforcement regarding fish‐bearing streams and associated buffers in the Pacific Northwest (Province of British Columbia, 2018) might be insufficient in mitigating the interference of human activities on the consumption of fitness‐related food for a sensitive large carnivore. For example, even under ‘ecosystem‐based management’ in regions of coastal BC, the minimum required riparian buffers of salmon‐bearing streams for forest harvest and road building are only 1–1.5 tree lengths (<~100 m; Province of British Columbia, 2016). In contrast, we identify strong patterns associated with disturbance within 1000 m of spawning areas. Regulations designed at larger spatial scales may benefit riparian management in BC and beyond, should safeguarding interactions between bears and salmon be a priority for managers (Mantyka‐Pringle et al., 2014; Sweeney & Newbold, 2014). Another management option to mitigate adverse effects is imposing temporal reduction in human activity (e.g., road closures during spawning; Whittington et al., 2019), which might allow bears to increase consumption. Similar landscape‐scale and access‐related interventions might help restore grizzly bears in areas where they are vulnerable or extirpated but still host abundant salmon (e.g., Fraser River drainages in Figure 1).

Our work also highlights the problems, challenges, and opportunities for cross‐jurisdictional management. For example, despite having profound influence on terrestrial communities, salmon in Canada are generally managed narrowly as a marine resource under federal jurisdiction (Darimont et al., 2010). In contrast, provinces manage terrestrial wildlife and resource extraction. The fact that these two levels of government rarely interact presents a central problem in cross‐ecosystem management (Artelle et al., 2016; Darimont et al., 2010). We note, however, some opportunities. Our results can directly inform ongoing ecosystem‐based management and cumulative effect assessments by Indigenous and provincial government agencies in coastal BC. This includes setting thresholds for the amount of disturbance permitted in areas of key importance for grizzly bears (e.g., restricting or excluding disturbance less than 1000 m from salmon‐bearing rivers) via revisions to the Great Bear Rainforest Order, which guides forestry practice for the region (Province of British Columbia, 2016). Additionally, Indigenous resource management agencies operating in coastal BC typically span marine, aquatic, and terrestrial systems, equipping them to address efficiently the threats to the maintenance of cross‐ecosystem linkages (Adams et al., 2021, 2023; Ban et al., 2018).

As biodiversity continues to decline worldwide, considerable debate ensues as to whether conservationists should prioritize the protection of areas unaffected by industrial development and intensify their efforts elsewhere, or prioritize less destructive development and agricultural practices across expansive areas to simultaneously address the needs of humans and nature. Here, we showed that the bear–salmon predator–prey system – an iconic, and formerly widespread, ecological interaction – appears sensitive to even low levels of human infrastructure and development. Accordingly, our results support calls to retain intact landscapes as a key strategy in mitigating biodiversity damage, especially for large vertebrates, from the expanding human footprint (Betts et al., 2017; Martin & Watson, 2016; Ramírez‐Delgado et al., 2022; Suraci et al., 2021; Watson et al., 2018). More broadly, increased consideration of how human activity might be managed spatially and temporally to reduce risk associated with focal species accessing key foods could benefit predator–prey associations and ecosystems globally.

## AUTHOR CONTRIBUTIONS

**Megan S. Adams:** Conceptualization (equal); data curation (equal); formal analysis (lead); methodology (equal); visualization (equal); writing – original draft (lead); writing – review and editing (lead). **Taal Levi:** Conceptualization (equal); formal analysis (equal); methodology (equal); supervision (supporting); writing – review and editing (supporting). **Mathieu Bourbonnais:** Formal analysis (equal); methodology (equal); visualization (equal); writing – review and editing (equal). **Christina N. Service:** Data curation (equal); formal analysis (equal); methodology (equal); visualization (equal); writing – review and editing (equal). **Kyle Artelle:** Data curation (equal); formal analysis (equal); methodology (equal); visualization (equal); writing – review and editing (equal). **Heather Bryan:** Data curation (equal); formal analysis (equal); methodology (equal); resources (equal); writing – original draft (equal). **Paul Paquet:** Conceptualization (equal); methodology (supporting); supervision (equal); writing – review and editing (equal). **Trisalyn Nelson:** Conceptualization (equal); formal analysis (supporting); methodology (supporting); supervision (equal); writing – review and editing (equal). **Chris T. Darimont:** Conceptualization (equal); funding acquisition (lead); methodology (equal); project administration (lead); supervision (lead); writing – review and editing (equal).

## CONFLICT OF INTEREST STATEMENT

The authors declare no conflict of interest.

### OPEN RESEARCH BADGES

This article has earned Open Data and Preregistered Research Designs badges. Data and the preregistered design and analysis plan are available at https://doi.org/10.5061/dryad.2fqz612w9.

## Supporting information


Appendix S1.

## Data Availability

Data are available from the Dryad Digital Repository at https://datadryad.org/stash/share/‐‐jvddanP‐xICUxbtkqaBotfbWGGRUPOSQcIuRDbZo4. Due to agreements with partnering Indigenous Nations, as well as the Province of British Columbia, precise sampling locations are not shared.
