# Supplementary material for: Human disturbance in riparian areas disrupts predator–prey interactions between grizzly bears and salmon
Source: Ecol Evol. 2024 Mar 19;14(3):e11058. doi: 10.1002/ece3.11058 (PMC10950355; doi:10.1002/ece3.11058)
Supplement: Supplementary file 1 — Appendix S1. [file ECE3-14-e11058-s001.docx]

Supplementary Information for: Human disturbance in riparian areas unravels predator-prey interactions between grizzly bears and salmon

**Supplementary Methods and Materials**

1. Estimating salmon consumption

*1.1 - Laboratory preparation*

Hair samples were washed and rinsed with a 2:1 mixture of chloroform:methanol to remove oils and surficial debris. After the samples were air dried for at least 48 hours, we cut each hair into small segments and subsampled ~1mg into tin capsules for continuous flow isotope ratio mass spectrom­­­­etry analysis. All isotope analysis was conducted at the stable isotope facility in the Department of Soil Sciences, University of Saskatchewan, Saskatoon, Canada using a Europa Scientific ANCA-NT gas-solid-liquid preparation module coupled to a Europa Scientific Tracer 20-20 mass spectrometer (PDZ Europa, Cheshire, England; (Darimont and Reimchen 2002, Reimchen and Klinka 2017).

All isotope ratios are expressed as δ values, which report parts per mil (%_o_), according to the equation:

δ*X*= ((R_sample_/R_standard_) – 1) x 1000

where *X* represents ^13^C or ^15^N, and R represents the ratio of heavy to light isotopes. Vienna-Pee Dee Belemnite limestone (V-PDB) and atmospheric N_2_ are the standard for carbon and nitrogen, respectively. The stable isotope laboratory at the University of Saskatoon estimates their analytical error at 0.05 (SD 0.04) for carbon, and 0.04 (SD 0.05) for nitrogen.

*1.2 - General modeling approach*

We estimated annual dietary contributions from predetermined food groups (Table S2) for individuals using MixSIAR (version 3.1.7, (Stock and Semmens 2013)) in R (version 3.5.1, (R Development Core Team 2018)). We structured all models with uninformative priors (whereby all combinations of dietary proportions were equally likely, based on a Dirichlet distribution (Stock and Semmens 2016b)), process error, and included each bear’s unique identification number as a random effect. Using Gibbs sampling for each model, we applied a burn-in of 200,000, a thinning interval of 100, and chain length of 300,000 across three chains. Model diagnostics (Gelman tests) confirmed satisfactory convergence of all models (Stock and Semmens 2013).

While a multiplicative error structure (process * residual) is recommended (Stock and Semmens 2016a), our models in the interior region failed to converge with multiplicative error. Given the high degree of omnivory and associated individual dietary specialization in bears (Mattson and Reinhart 1995, Lafferty et al. 2015), and with the potential for sampling error across a large study area, we structured both interior and coastal models instead with process error (see Deacy et al. (2018), who used a similar model structure in a coastal ecosystem, (Deacy et al. 2018)). We note that diagnostics and model selection criteria (DIC) supported using process error over residual error (but see (Stock and Semmens 2016a)).

*1.3 - Major food groups and isotope source values*

We modeled annual proportions of food group contributions by including only foods consumed in the regions in which individuals were sampled (Hopkins and Kurle 2016, Adams et al. 2017). We determined prey sample isotope values from the literature. We included values from source samples that were originally collected from regions appropriate to the spatial scale and ecological context of our sampled bear hair (see Table S2) (Mowat and Heard 2006, Adams et al. 2017). We confirmed suitable isotopic mixing space geometry by plotting raw stable isotope carbon and nitrogen values (δ^13^C, δ^15^N) for each grizzly bear hair sample alongside the mean (± 1SD) carbon and nitrogen isotopic source values (corrected for isotopic discrimination: ∆^13^C, ∆^15^N) for food groups in interior and separately in coastal regions (Table S2).

*1.4 - Trophic discrimination factors*

Given an absence of empirically-estimated discrimination factors for bear hair (Hopkins and Kurle 2016), and following Hopkins and Kurle (2016), we used discrimination values from laboratory-controlled feeding experiments of Norway rats - a similarly omnivorous mammal (Table S2) (Hopkins and Kurle 2016, Hopkins et al. 2017, Service et al. 2018).

*1.5 - Digestibility*

We explored incorporating concentration dependence into our mixing models to account for the omnivorous nature of grizzly bears, which consume a range of food items that vary in C:N ratios (Phillips and Koch 2002). We assessed whether to carry our concentration independent or concentration dependent model results into our main analysis using leave-one-out cross validation (LOO) (Stock and Semmens 2016a, Vehtari et al. 2017) and DIC, which has been more broadly applied in Bayesian model selection (Table S3) (Hopkins et al. 2017). We applied concentration values derived from digestibility from previous studies that focused on the same food items bears in our system consume (Table S2) (Fox et al. 2014, Hopkins et al. 2017, Service et al. 2018, Deacy et al. 2018). When concentration dependence was used, we used digestible elemental concentration values following Hopkins et al. (2017) and Deacy et al. (2018) (plant matter: = C = 45 + 0, N = 5.84 + 3.70, n = 43; deer (used for terrestrial meat): C = 51.5 + 0, N = 15.5 +0.54, n = 4; trout (*O. mykiss*): C = 54.8 + 0 , N = 11.65 + 4.12, n = 6, where the trout value was used for salmon, given it yielded identical effects in a previous study for bears from a similar coastal temperate ecosystem (use in (Deacy et al. 2018)). As we could find no previously calculated N digestibility in the literature for intertidal animals, we calculated values from an intertidal organism commonly consumed by bears (*Hemigrapsus nudis*), for which data were available. We applied previously calculated C digestibility values specific to animal sources (n = 18; C = 51.5 + 0, N = 15.6 + 0.83; (Fox et al. 2014)).

We determined that our concentration independent model was most appropriate for our data based on both LOO and DIC values. Importantly, the individual’s salmon consumption values between the concentration independent and dependent model were highly positively correlated (Pearson’s r = 0.987 for coastal model, r = 0.997 for interior model). The concentration independent model was also the most appropriate given the inherent uncertainty associated with concentration dependent models in estimating the relative contributions and digestibility of food groups, especially plants (Table S3) (Deacy et al. 2018). As such, although mean dietary salmon estimates were slightly lower from the concentration dependent model (Table S3), salmon estimates derived from both models should have near identical relationships with the predictor variables. For these reasons, in addition to the added uncertainty in estimating the digestibility and relative contributions of plants to bear diets across our large study over multiple years (Robbins et al. 2002, Deacy et al. 2018), we were confident proceeding with the output from our concentration-independent model. In addition, we wanted to compare our results to previous results in BC and elsewhere that use concentration independent models (e.g., (Service et al. 2018, Deacy et al. 2018)).

*1.6 - Limitations*

We carried forward only median estimates of the proportion of annual dietary salmon contributions into our GLMM framework to test predictions related to covariates affecting salmon consumption. This approach ignores associated uncertainty from the posterior distribution for each individual estimate. Specifically, in our dataset the mean standard deviation of coast (mean SD = 0.071) and interior (mean SD = 0.077) individual dietary salmon estimates was not considered in our analysis structure. We used this simplified approach to reduce modeling complexity­­ but acknowledge the limitations that it may impose on inference. However, we note that our median estimate for the coast and interior models were highly correlated to both the lower bound of the 95% credible interval estimate from each individual’s posterior (Pearson r = 0.980, r = 0.983, respectively) as well as the upper bound (Pearson r = 0.983, r = 0.991, respectively). This high correlation suggests that the patterns we observed would hold at both the upper and lower bounds of each individual’s posterior distribution of annual dietary salmon consumption.

1. Estimating salmon availability

We estimated yearly salmon biomass (‘biomass’) and species diversity (‘diversity’) available to grizzly bears in each watershed using salmon enumeration data from Fisheries and Oceans Canada (FOC 2016). These data represent an index subset of all salmon-bearing waterways in British Columbia. Geographic coordinates for salmon enumeration points were not included in the 2016 version of the data, so we joined the 2016 data with coordinates from a previous version using a unique stream identifier (FOC 2016). We excluded enumeration points without a geographic location (n =137 of 2137). Although we used the most recent data available at the time of our analyses, with records current to 2013, only 8% of runs are monitored consistently (Price et al. 2008, 2017). To account for missing enumeration data for these monitored streams, we applied an imputation method following Ruggerone et al. (2010) and Bryan et al. (2014) (Ruggerone et al. 2010, Bryan et al. 2014). This method imputes missing data using the average contribution of a particular stream and salmon species over the 20-year study period relative to the average total abundance for that species across all streams in the same watershed. The missing value is calculated by multiplying the average annual contribution for that species and stream by the average performance of other streams from the same watershed in that year relative to their respective average annual contributions. As such, the imputation method assumes that nearby streams perform similarly relative to their long-term average salmon abundance.

Where possible, we used salmon abundance estimates from other streams in the same third-order watershed to impute missing species-year-stream estimates. In watersheds with enumeration points for less than five streams, we grouped streams in adjacent watersheds that were upstream or downstream of the sparsely counted watershed. In coastal areas, if there were no other well-populated watersheds upstream or downstream, we chose the closest adjacent well-populated watershed with the nearest ocean outlet. To be eligible for imputation, we required that each stream and species have estimates for a minimum of 14 (70%) of the 20 years in our study period, with at least 3 (15%) observations occurring in the latter 10 years. For pink salmon with even- and odd-year runs—which we considered separate species due to their distinct two-year lifecycles—we required at least 7 observations in the 20-year study period, with at least one in the latter 10 years. After applying these exclusion criteria, 10% of 15,500 possible abundance species-year-stream estimates were eligible for imputation over the 20-year study period.

Following the imputations, we used ArcGIS 10.2.2 (ESRI, Redlands, California, USA) and Python 2.7.5 (http://www.python.org) to join salmon enumeration points to their corresponding spawning salmon stream line segments (BCGOV 2005). Where possible, we joined enumeration to spawning reaches of streams only (BCGOV 2006). If no corresponding lines existed, we joined enumeration points to line segments from a spatial dataset of streams covering the entire province of BC (BCGOV 2005). We then calculated salmon biomass in each watershed by multiplying abundance estimates by the average mass of salmon (kg) and summing across species, based on average mass of both sexes and a 1:1 sex ratio (Groot and Margolis 1991). We intersected watersheds with salmon streams and calculated the length of each stream segment within a watershed. In R, we calculated the total salmon biomass per watershed and year using the ratio of total biomass for a given salmon species to total stream length and multiplying by the length of the stream segment within the watershed. We then summed biomass across all species and stream segments within each watershed in a given year and divided the total biomass estimate by the useable area of the watershed to compute an estimated salmon biomass density within watersheds. Finally, we assigned a salmon biomass estimate to each bear based on its watershed of origin and year hair was grown.

We log transformed salmon biomass density, reasoning that the odds of a bear consuming salmon are more sensitive to proportional changes in abundance than linear changes (i.e. 100 additional kilograms of salmon means more to a bear where salmon are rare than where salmon are common) (Bryan et al. 2014, Erlenbach et al. 2014). This transformation also improved the normality of the covariate.

Increased species diversity can result in spatiotemporal asynchrony of salmon availability that we reasoned would extend foraging opportunities for bears (Schindler et al. 2013, Service et al. 2018). Complementing our estimates of salmon biomass density, we used the Shannon diversity index to estimate salmon diversity in each watershed:

$$\left( 1 \right) H=-\sum_{i=1}^{S} p_{i}ln(p_{i})$$

where *S* is the number of salmon species and p*_i_* is the proportion of the total estimated salmon biomass comprising the *i*^th^ species. We used biomass estimates instead of enumeration counts to estimate diversity, because we reasoned that total biomass would be more relevant to bear diets (for example, on average pink salmon (even year) weigh 1.7 kg, while Chinook salmon weighs 13.6 kg; (Groot and Margolis 1991)). Accounting for both species richness and evenness in species abundance, the Shannon diversity index provides a proxy for salmon resource availability over space and time.

1. GLMM Model diagnostics

We examined potential collinearity among covariates that might occur due to regional (i.e., coastal versus interior) trends. We assigned watersheds to either coastal (n = 12) or interior (n = 10) regions as delineated by the boundary of BC’s ‘coast and mountain temperate rainforest’ eco-province (Demarchi 2011). We considered the full set of candidate models (Table S4) with and without a region factor included. We found the same top models as our original analysis, which did not include region as a factor (Table 1). The direction and magnitude of parameter estimates in the top model set did not differ with region included. Accordingly, we did not include the region factor in our analysis. Post-hoc examination for multicollinearity among predictor variables for model sets with and without region, inspected with the *car* package (version 3.2.0 in R, (Fox and Weisberg 2018)), indicated low variance inflation factors (~ 1 for all parameters).

Standardized coefficient estimates suggest the negative association with footprint was stronger than the positive association with salmon biomass density. To assess the robustness of this result, we performed additional post-hoc analyses to explore the potential impacts of salmon measurement error by the inclusion of additional variables in our candidate models. We reasoned that salmon measurement effort and/or error increases towards coastal watersheds where streams are smaller and less-commercially viable species spawn (Price et al. 2008, Service et al. 2018). Unlike large and commercially valuable species like sockeye or chinook, which often travel hundreds of kilometres into interior rivers, other salmon species that spawn in coastal areas often receive less enumeration effort (Price et al. 2008). We assessed distance to coast for each bear detection and calculated the proportion of enumerated populations per watershed as a proxy of salmon measurement effort. We hypothesized that salmon consumption would increase as distance to the coast decreased because coastal bears have greater access to prolonged salmon foraging opportunities due to the increased density and diversity of salmon species in coastal watersheds. We hypothesized that salmon consumption would decrease with increased enumeration effort, because coastal watersheds with higher salmon diversity across a greater number of small streams receive less effort than large, less diverse salmon populations in the interior (Price et al. 2008, Service et al. 2018).

We calculated Euclidean distance to the BC coastline (in QGIS 3.4, (Map Cruzin 2019)) for each bear detection location. In addition, we calculated the number of populations enumerated from the index subset of all salmon populations from Fisheries and Oceans Canada (FOC 2016), compared with the total number of identified salmon populations (BCGOV 2006) for each watershed. We considered a population to have enumeration effort if count data existed for that population within the years of our analysis. Mean proportion of enumeration for watersheds in the coastal region was roughly half of interior watersheds (coastal mean = 0.17, interior mean = 0.32).

After calculating these additional two variables, we competed our original set of 25 candidate models (Table S4) against the same model set where models 2-25 also included Euclidean distance to the coast and proportion of enumerated populations. The top models that emerged were the same as in our original comparison and did not contain the new variables, indicating that measurement effort and distance to coast were not important (Table 1). Of those models that included the new variables, the coefficients of both were negative (aligning with hypotheses), although not strong in effect size (i.e., CIs overlapped zero).

**Fig. S1.** Example watershed (Upper Fraser River watershed basin, containing the town of Valemount, British Columbia, Canada): Spawning salmon reaches are shown in blue, and early seral forest areas are shown in green. Climate variables (mean growing season temperature and precipitation; see full details and supporting references in Methods) were sampled annually at 4 km intervals in habitable areas (light grey). The inset shows the area within the rectangle, with a 1 km buffer on either side of the spawning reach that intersects with human footprint index (HFI) raster pixels (1x1 km).

**Fig. S2.** Spatial distribution of covariates within watersheds - (a) growing season temperature, (b) growing season precipitation, (c) proportion of watershed area of early seral (<10 yr old) forest, (d) salmon species diversity, (e) salmon biomass density, and (f) human footprint index - used to predict annual proportions of salmon to grizzly bear (*U. arctos horribilis*) diets across British Columbia, Canada from 1995 to 2014. Means represent covariate data from years in which bear samples were collected.

**Fig. S3.** Residuals of top GLMMs (see Table S4 & S5) used to predict annual proportion of dietary salmon in diets of grizzly bears (*Ursus arctos horribilis*) across BC (1995-2014).

**
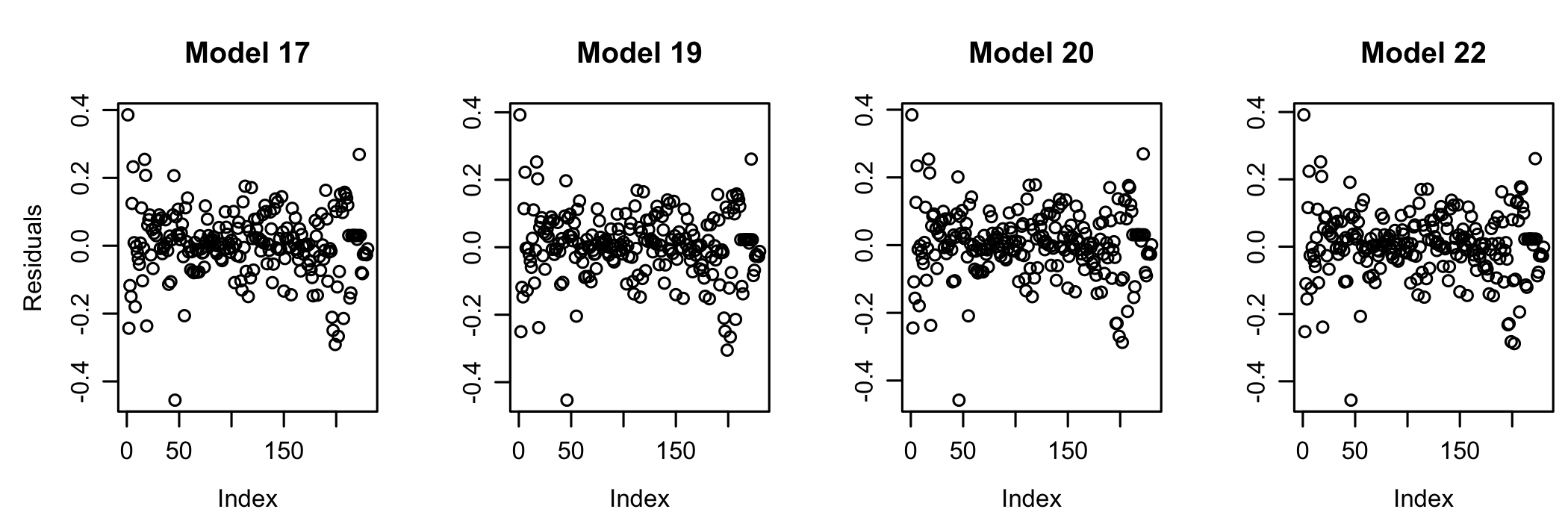
**

**Table S1.** Home range sizes for adult male and female grizzly bears in interior and coastal habitats in western North America.

| **Region** | **Males (mean area, km^2^)** | **Females (mean area, km^2^)** | **Sources** |
| --- | --- | --- | --- |
| Flathead valley | 446 (n = 5) | 200 (n = 5) | LeFranc et al. 1987, Hatler et al. 2008 |
| Interior BC (Revelstoke) | 279 (n = 7) | 79 (n = 3) | LeFranc et al. 1987, Pasitschniak-Arts 1993, Hatler et al. 2008 |
| NW Montana (Yellowstone) | 828 (n = NA) | 384 (n = NA) | LeFranc et al. 1987, Pasitschniak-Arts 1993 |
| Jasper National Park | 916 (n = 11) | 244 (n = 2) | LeFranc et al. 1987, Pasitschniak-Arts 1993 |
| SW Alaska (coastal) | NA | 357 (n = 40) | Collins et al. 2005 |
| Khutzeymateen Valley (coastal) | 130 (n = 4) | 52 (n = 13) | MacHutchon et al. 1993 |

**Table S2.** Adapted from Adams et al. (2017)(Adams et al. 2017). Isotope ratios (δ^13^C and δ^15^N) used to calculate annual proportions of salmon to grizzly bear (*U. arctos horribilis*) diets across British Columbia, Canada (1995-2014). Shown are the applied isotopic baselines for food items (e.g., δ^13^C) and trophic discrimination values from food to bear hair (e.g., ∆^13^C).

| **Food type** | **δ^13^C**  **(‰; SD)** | ∆^13^C **(SD)** | **δ^15^N**  **(‰; SD)** | ∆^15^N **(SD)** | **n^†^** | **Sources** |
| --- | --- | --- | --- | --- | --- | --- |
| Terrestrial Meat | | | | | | |
| *Coast: black tailed deer* | -24.8  (1.0) | 2.1  (0.1) | 2.5  (1.0) | 3.9  (0.1) | 107 | Jacoby et al. 1999, Szepanski et al. 1999, Ben-David et al. 2004, Mowat and Heard 2006 |
| *Interior: moose, elk, and white-tailed and mule deer* | -25.3  (1.0) | 2.1  (0.1) | 3.4  (1.0) | 3.9  (0.3) | 70 | Jacoby et al. 1999, Hobson et al. 2000, Felicetti et al. 2005, Mowat and Heard 2006 |
| Generalized salmon baseline | -19.9  (1.0) | 2.1  (0.1) | 12.5  (1.0) | 3.9  (0.3) | 338 | Bilby et al. 1996, Hilderbrand et al. 1996, Jacoby et al. 1999, Satterfield and Finney 2002, Ben-David et al. 2004; G. Mowat personal communication 2015 |
| Generalized intertidal baseline | -14.9  (1.41) | 2.1  (0.1) | 8.67  (1.17) | 3.9  (0.3) | 16 | Ben-David et al. 2004, Fox et al. 2014 |
| Generalized plants baseline | -26.6  (2.0) | 3.4  (0.5) | -2.8  (3.0) | 2.4  (0.2) | 200 | Mowat and Heard 2006 |

n**^†^** This sample size applies to both carbon and nitrogen.

**Table S3.** Assimilated dietary estimates and associated model selection criteria (LOO and DIC) of concentration-independent and concentration dependent models for coastal and interior grizzly bears in British Columbia, Canada, from 1995-2014. Population level model dietary proportion values include: Mean, 1 SD, Median, and 95% CI (credible interval). Range denotes the range of mean proportion estimates among individual bear-year combinations.

|  | Concentration Independent | | | | | Concentration Dependent | | | | |
| --- | --- | --- | --- | --- | --- | --- | --- | --- | --- | --- |
| *Dietary Item* | *Mean* | *SD* | *Median* | *95% CI* | *Range* | *Mean* | *SD* | *Median* | *95% CI* | *Range* |
| ***Coast***  *Model selection criteria:* LOO = -152.2, DIC = 9.036 | | | | | | LOO = -123.5, DIC = 118.435 | | | | |
| *Intertidal* | 0. | 0. | 0. | 0.-0. | 0.-0. | 0. | 0. | 0. | 0.-0. | 0.-0. |
| *Plants* | 0. | 0. | 0. | 0.-0. | 0.-0. | 0. | 0. | 0. | 0.-0. | 0.-0. |
| *Terrestrial Meat* | 0. | 0. | 0. | 0.-0. | 0.-0. | 0. | 0. | 0. | 0.-0. | 0.-0. |
| *Salmon* | 0. | 0. | 0. | 0.-0. | 0.-0. | 0. | 0. | 0. | 0.-0. | 0.-0. |
| ***Interior***  *Model selection criteria:* LOO = 32.5, DIC = 41.469 | | | | | | LOO = 58.4, DIC = 64.524 | | | | |
| *Plants* | 0. | 0. | 0. | 0.-0. | 0.-0. | 0. | 0. | 0. | 0.-0. | 0.-0. |
| *Terrestrial Meat* | 0. | 0. | 0. | 0.-0. | 0.-0. | 0. | 0. | 0. | 0.-0. | 0.-0. |
| *Salmon* | 0. | 0. | 0. | 0.-0. | 0.-0. | 0. | 0. | 0. | 0.-0. | 0.-0. |

**Table S4.** Candidate model set used to assess the effect of ecological variables on annual proportion of salmon in diet of grizzly bears across British Columbia from 1995-2014. We predicted that males consume more salmon than females (Adams et al. 2017), and thus sex appears in all our models. All models included year and watershed as random effects. Top models (ΔAIC > 2, (Burnham and Anderson 2002)) are in grey.

| **Model** | **Fixed effects** | | **ΔAIC** | | **Df** | **Weight** |
| --- | --- | --- | --- | --- | --- | --- |
| 1 | Intercept-only | | 114.2 | | 4 | <0.001 |
| **H1 – Salmon availability** | |  |  |  |  |  |
| 2 | biomass + diversity | | 50.6 | | 7 | <0.001 |
| 3 | biomass + diversity + biomass*diversity | | 50.6 | | 8 | <0.001 |
| 4 | biomass + diversity + biomass*diversity + biomass*sex + diversity*sex | | 54.5 | | 10 | <0.001 |
| **H2 – Salmon availability & alternative food** | | |  | |  |  |
| 5 | biomass + diversity + precip + temp + earlystage | | 19.7 | | 10 | <0.001 |
| 6 | biomass + diversity + biomass*diversity + precip + temp + earlystage | | 20.5 | | 11 | <0.001 |
| 7 | biomass + diversity + biomass*diversity + biomass*sex + diversity*sex + precip + temp + earlystage | | 24.2 | | 13 | <0.001 |
| **H3 – Salmon availability & human footprint** | |  |  |  |  |  |
| 8 | biomass + diversity + footprint | | 28.6 | | 8 | <0.001 |
| 9 | biomass + diversity + footprint + biomass*footprint + diversity*footprint | | 31.4 | | 10 | <0.001 |
| 10 | biomass + diversity + footprint + sex*footprint | | 28.6 | | 9 | <0.001 |
| 11 | biomass + diversity + biomass*diversity + footprint | | 28.7 | | 9 | <0.001 |
| 12 | biomass + diversity + biomass*diversity + footprint + biomass*footprint + diversity*footprint | | 31.2 | | 11 | <0.001 |
| 13 | biomass + diversity + biomass*diversity + footprint + sex*footprint | | 28.7 | | 10 | <0.001 |
| 14 | biomass + diversity + biomass*diversity + biomass*sex + diversity*sex + footprint | | 32.0 | | 11 | <0.001 |
| 15 | biomass + diversity + biomass*diversity + biomass*sex + diversity*sex + footprint + biomass*footprint + diversity*footprint | | 33.4 | | 12 | <0.001 |
| 16 | biomass + diversity + biomass*diversity + biomass*sex + diversity*sex + footprint + sex*footprint | | 32.2 | | 12 | <0.001 |
| **H3 - Salmon availability & alternative food & human footprint** | | | |  |  |  |
| 17 | biomass + diversity + footprint + precip + temp + earlystage | | 0.0 | | 11 | 0.255 |
| 18 | biomass + diversity + footprint + biomass*footprint + diversity*footprint + precip + temp + earlystage | | 2.7 | | 13 | 0.066 |
| 19 | biomass + diversity + footprint + sex*footprint + precip + temp + earlystage | | 0.4 | | 12 | 0.207 |
| 20 | biomass + diversity + biomass*diversity + footprint + precip + temp + earlystage | | 0.7 | | 12 | 0.181 |
| 21 | biomass + diversity + biomass*diversity + footprint + biomass*footprint + diversity*footprint + precip + temp + earlystage | | 3.1 | | 14 | 0.055 |
| 22 | biomass + diversity + biomass*diversity + footprint + sex*footprint + precip + temp + earlystage | | 0.9 | | 13 | 0.164 |
| 23 | biomass + diversity + biomass*diversity + biomass*sex + diversity*sex + footprint + precip + temp + earlystage | | 4.4 | | 14 | 0.028 |
| 24 | biomass + diversity + biomass*diversity + biomass*sex + diversity*sex + footprint + biomass*footprint + diversity*footprint + precip + temp + earlystage | | 4.9 | | 15 | 0.022 |
| 25 | biomass + diversity + biomass*diversity + biomass*sex + diversity*sex + footprint + sex*footprint + precip + temp + earlystage | | 4.8 | | 15 | 0.023 |

**Supplementary References**

Adams, M. S., C. N. Service, A. Bateman, M. Bourbonnais, K. A. Artelle, T. Nelson, P. C. Paquet, T. Levi, and C. T. Darimont. 2017. Intrapopulation diversity in isotopic niche over landscapes: Spatial patterns inform conservation of bear-salmon systems. Ecosphere 8:e01843.

Artelle, K. A., S. C. Anderson, J. D. Reynolds, A. B. Cooper, P. C. Paquet, and C. T. Darimont. 2016. Ecology of conflict: marine food supply affects human-wildlife interactions on land. Scientific Reports 6:25936.

BCGOV. 2005. WSA-Stream centreline network 50k spatial dataset. Ministry of Environment and Climate Change Strategy, British Columbia Ministry of Environment. https://catalogue.data.gov.bc.ca/dataset/wsa-stream-centreline-network-50-000/resource/3d01e2b9-57d6-4fa1-a603-876b56102536.

BCGOV. 2006. The British Columbia historical fish distribution 50k spatial dataset. Ministry of Environment and Climate Change Strategy, British Columbia Ministry of Environment. http://www.env.gov.bc.ca/esd/distdata/ecosystems/bc50kfiss/hist_fish_dist/.

BCGOV. 2011. WSA-BC watershed groups 50k spatial dataset. Ministry of Environment and Climate Change Strategy, British Columbia Ministry of Environment. https://catalogue.data.gov.bc.ca/dataset/wsa-bc-watershed-groups-50-000.

Ben-David, M., K. Titus, and L. R. Beier. 2004. Consumption of salmon by Alaskan brown bears: a trade-off between nutritional requirements and the risk of infanticide? Oecologia 138:465–474.

Bilby, R. E., B. R. Fransen, and P. A. Bisson. 1996. Incorporation of nitrogen and carbon from spawning coho salmon into the trophic system of small streams: evidence from stable isotopes. Canadian Journal of Fisheries and Aquatic Sciences 53:164–173.

Bryan, H. M., C. T. Darimont, P. C. Paquet, K. E. Wynne-Edwards, and J. E. Smits. 2014. Stress and reproductive hormones reflect inter-specific social and nutritional conditions mediated by resource availability in a bear–salmon system. Conservation Physiology 2:cou010.

Burnham, K. P., and D. R. Anderson. 2002. Model selection and multimodel inference: a practical information-theoretic approach. 2nd ed. Springer, New York, NY.

Collins, G. H., S. D. Kovach, and M. T. Hinkes. 2005. Home range and movements of female brown bears in Southwestern Alaska. Ursus 16:181–189.

Darimont, C. T., and T. E. Reimchen. 2002. Intra-hair stable isotope analysis implies seasonal shift to salmon in gray wolf diet. Canadian Journal of Zoology 80:1638–1642.

Deacy, W. W., J. A. Erlenbach, W. B. Leacock, J. A. Stanford, C. T. Robbins, and J. B. Armstrong. 2018. Phenological tracking associated with increased salmon consumption by brown bears. Scientific Reports 8:11008.

Demarchi, D. A. 2011. The British Columbia Ecoregion Classification. Ecosystem Information Section, Ministry of Environment, Victoria, BC.

Erlenbach, J. A., K. D. Rode, D. Raubenheimer, and C. T. Robbins. 2014. Macronutrient optimization and energy maximization determine diets of brown bears. Journal of Mammalogy 95:160–168.

Felicetti, L. A., C. T. Robbins, S. Herrero, and M. Pinto. 2005. Diet of some eastern slopes grizzly bear project bears as determined by stable isotope analysis. University of Calgary, Calgary, AB.

FOC. 2016. NuSEDS regional adult salmon escapement database 1950-2016. Pacific Biological Station, Fisheries and Oceans Canada, Nanaimo, BC.

Fox, C. H., R. El-Sabaawi, P. C. Paquet, and T. E. Reimchen. 2014. Pacific herring *Clupea pallasii* and wrack macrophytes subsidize semi-terrestrial detritivores. Marine Ecology Progress Series 495:49–64.

Fox, J., and S. Weisberg. 2018. An R Companion to Applied Regression. SAGE Publications, Thousand Oaks, CA.

Groot, C., and L. Margolis. 1991. Pacific Salmon Life Histories. UBC Press, Vancouver, BC.

Hatler, D. F., D. W. Nagorsen, and A. M. Beal. 2008. Carnivores of British Columbia. Royal BC Museum, Victoria, BC.

Hilderbrand, G. V., S. D. Farley, C. T. Robbins, T. A. Hanley, K. Titus, and C. Servheen. 1996. Use of stable isotopes to determine diets of living and extinct bears. Canadian Journal of Zoology 74:2080–2088.

Hobson, K. A., B. N. McLellan, and J. G. Woods. 2000. Using stable carbon (δ13C) and nitrogen (δ15N) isotopes to infer trophic relationships among black and grizzly bears in the upper Columbia River basin, British Columbia. Canadian Journal of Zoology 78:1332–1339.

Hopkins, J. B., J. M. Ferguson, D. B. Tyers, and C. M. Kurle. 2017. Selecting the best stable isotope mixing model to estimate grizzly bear diets in the Greater Yellowstone Ecosystem. PLoS One 12:e0174903.

Hopkins, J. B., and C. M. Kurle. 2016. Measuring the realized niches of animals using stable isotopes: from rats to bears. Methods in Ecology and Evolution 7:210–221.

Jacoby, M. E., G. V. Hilderbrand, C. Servheen, C. C. Schwartz, S. M. Arthur, T. A. Hanley, C. T. Robbins, and R. Michener. 1999. Trophic relations of brown and black bears in several western North American ecosystems. The Journal of Wildlife Management:921–929.

Lafferty, D. J. R., J. L. Belant, and D. L. Phillips. 2015. Testing the niche variation hypothesis with a measure of body condition. Oikos 124:732–740.

LeFranc, M. Jr., M. Moss, K. Patnode, and W. Sugg III, editors. 1987. Grizzly bear compendium. International Grizzly Bear Committee, Washington DC, USA.

MacHutchon, A. G., S. Himmer, and C. A. Bryden. 1993. Khutzeymateen Valley grizzly bear study: final report. Wildl. Rep., Ministry of Environment, Lands, and Parks and Ministry of Forests, Government of British Columbia, Victoria, BC.

Map Cruzin. 2019. British Columbia ArcGIS GIS Shapefile Map Layers via Open Stree Map. https://mapcruzin.com/free-canada-british-columbia-arcgis-maps-shapefiles.htm.

Mattson, D. J., and D. P. Reinhart. 1995. Influences of cutthroat trout (*Oncorhynchus clarki*) on behaviour and reproduction of Yellowstone grizzly bears (*Ursus arctos*), 1975 – 1989. Canadian Journal of Zoology 73:2072–2079.

Mowat, G., and D. C. Heard. 2006. Major components of grizzly bear diet across North America. Canadian Journal of Zoology 84:473–489.

Pasitschniak-Arts, M. 1993. *Ursus arctos*. Mammalian Species:1–10.

Phillips, D. L., and P. L. Koch. 2002. Incorporating concentration dependence in stable isotope mixing models. Oecologia 130:114–125.

Price, M. H. H., C. T. Darimont, N. F. Temple, and S. M. MacDuffee. 2008. Ghost runs: management and status assessment of Pacific salmon (*Oncorhynchus* spp.) returning to British Columbia’s central and north coasts. Canadian Journal of Fisheries and Aquatic Sciences 65:2712–2718.

Price, M. H. H., K. K. English, A. G. Rosenberger, M. MacDuffee, and J. D. Reynolds. 2017. Canada’s Wild Salmon Policy: an assessment of conservation progress in British Columbia. Canadian Journal of Fisheries and Aquatic Sciences 74:1507–1518.

R Development Core Team. 2018. R: A language and environment for statistical computing. R Foundation for Statistical Computing, Vienna, Austria.

Reimchen, T. E., and D. R. Klinka. 2017. Niche differentiation between coat colour morphs in the Kermode bear (Ursidae) of coastal British Columbia. Biological Journal of the Linnean Society 122:274–285.

Robbins, C. T., G. V. Hilderbrand, and S. D. Farley. 2002. Incorporating concentration dependence in stable isotope mixing models: a response to Phillips and Koch (2002). Oecologia 133:10–13.

Ruggerone, G. T., R. M. Peterman, B. Dorner, and K. W. Myers. 2010. Magnitude and trends in abundance of hatchery and wild pink salmon, chum salmon, and sockeye salmon in the North Pacific Ocean. Marine and Coastal Fisheries 2:306–328.

Satterfield, F. R., and B. P. Finney. 2002. Stable isotope analysis of Pacific salmon: insight into trophic status and oceanographic conditions over the last 30 years. Progress in Oceanography 53:231–246.

Schindler, D. E., J. B. Armstrong, K. T. Bentley, K. Jankowski, P. J. Lisi, and L. X. Payne. 2013. Riding the crimson tide: mobile terrestrial consumers track phenological variation in spawning of an anadromous fish. Biology Letters 9:20130048.

Service, C. N., A. W. Bateman, M. S. Adams, K. A. Artelle, T. E. Reimchen, P. C. Paquet, and C. T. Darimont. 2018. Salmonid species diversity predicts salmon consumption by terrestrial wildlife. Journal of Animal Ecology 88:392–404.

Stock, B. C., and B. X. Semmens. 2013. MixSIAR GUI User Manual, version 3.0.

Stock, B. C., and B. X. Semmens. 2016a. Unifying error structures in commonly used biotracer mixing models. Ecology 97:2562–2569.

Stock, B., and B. Semmens. 2016b. MixSIAR GUI User Manual v3.1:59.

Szepanski, M. M., M. Ben-David, and V. Van Ballenberghe. 1999. Assessment of anadromous salmon resources in the diet of the Alexander Archipelago wolf using stable isotope analysis. Oecologia 120:327–335.

Vehtari, A., A. Gelman, and J. Gabry. 2017. Practical Bayesian model evaluation using leave-one-out cross-validation and WAIC. Statistics and Computing 27:1413–1432.
